# Radiology in conflict: scoping review

**DOI:** 10.1186/s13031-023-00550-9

**Published:** 2024-01-19

**Authors:** Trisha Suji, Richard Sullivan, Gemma Bowsher

**Affiliations:** 1 School of Medicine, King’s College London, SE1 9RT London Bridge, UK; 2 Centre for Conflict & Health Research, King’s College London, WCR 2LS Strand, UK

**Keywords:** Radiology, Imaging, MRI, CT, X-ray, PET, Ultrasound, Conflict, Military, War

## Abstract

The United Nations estimate a quarter of the global population currently lives in violent conflict zones. Radiology is an integral part of any healthcare system, providing vital information to aid diagnosis and treatment of a range of disease and injury. However, its delivery in conflict-affected settings remains unclear. This study aims to understand how radiology services are currently delivered in conflict settings, the challenges of doing so, and potential solutions. A hermeneutic narrative review of multiple databases, including grey literature sources, was undertaken. Key themes were identified, and articles grouped accordingly. Various conflict zones including Gaza, Ukraine, Iraq, Yemen, Afghanistan, and Somalia were identified in literature relating to radiology services. Three key themes were identified: underserving of local medical imaging services, strong presence of military hospitals, and the importance of teleradiology. A severe shortage of radiologists, technicians, and equipment in conflict affected settings are a significant cause of the underserving by local services. Teleradiology has been used to blunt the acuity of the these struggling services, alongside military hospitals which often serve local populations. Radiology faces unique challenges compared to other healthcare services owing to its expensive equipment which is difficult to fund and can be less effective due to international sanctions placed on contrast medium to enhance image quality. Further the equipment is reliant on local infrastructure, e.g., power supply, which can be affected in conflict. Key recommendations to improve radiology services include retention of radiologists within conflict zones, careful allocation of funds to supply necessary imaging machinery, international cooperation to ensure sanctions do not affect sourcing of radiology equipment, special training for military medical teams to help preparedness for the unique demands of the local population, and investment in communication devices, like smartphones, to allow international teleradiology efforts.

## Key message

Medical imaging services are critical element of a health system that is particularly constrained in conflict-affected settings.

Much of the medical imaging provided in conflict is delivered by external or remote groups including militaries and humanitarian medical communities.

The radiology workforce is very limited in conflict-affected settings – the exodus of health workers and limited training opportunities hamper further workforce development.

Technological requirements are limiting the full expansion of imaging services as conflict-affected health sectors experience continued degradation of key infrastructure including information technology required to sustain services.

Compliance with international regulatory frameworks for radiological safety are essential, yet even more complex in conflict-affected settings.

## Introduction

Delivering healthcare in conflict-affected settings produces unique health sector challenges. Radiology encompasses many different imaging techniques including X-ray, Computer Tomography (CT), Magnetic Resonance Imaging (MRI), and Nuclear imaging techniques such as Positron Emission Tomography (PET) and Single Photon Computer Emission Tomography (SPECT). These key words are explained in Table 1. Robust radiology services are essential for accurate diagnosis and treatment of a range of disease and injury – helping to ensure safe and efficient healthcare.

Conflict health systems are tasked with addressing both the immediate consequences of war-related injury and disease, and the regular population health burden across communicable, non-communicable and trauma domains. The United Nations currently estimate that one quarter of humanity, or two billion people, live in a conflict-affected setting [1]. Therefore, the demand on healthcare services, including radiology, in hostile environments is high. Furthermore, conflict-affected States undergo significant fiscal changes that degrade their ability to finance endogenous equipment [2].

The recent Lancet Commission on Diagnostics explored global access to radiology services [2]. It found that 47% of the global population have little to no access to these diagnostics, with this discrepancy disproportionately manifesting in low- and middle-income countries where just 19% have access to simple diagnostic test in primary care and 60–70% for secondary care [2, 3]. They identified 1.1 million annual deaths preventable deaths should access be improved, demonstrating the cruciality of these services [2].The biggest cause of the global paucity of diagnostic services was deemed its relatively low visibility with decision-makers, which leads to poor investment. The main operational barriers were linked to the adequacy of physical infrastructure [2]. Importantly, the Commission specifically noted the challenge of delivering diagnostic services in conflict environments [2].

This review aims to improve the understanding of how radiology services have been delivered in conflict zones. Subsequently, areas of best practise and those requiring innovation can be highlighted to support health sector planning and system-strengthening efforts.

## Methods

This study was carried out via a narrative hermeneutic review methodology to examine the role of radiology and imaging services in conflict settings [4].

Due to the often disparate nature of clinical and health system research in conflict, a narrative search methodology was deemed appropriate in order to capture the heterogeneity of published evidence, information sources and knowledge communities.

Articles were screened according to eligibility according to the “population, intervention, comparison, outcome” (PICO) format. Sources were included if they described radiology services operating in a conflict-affected setting and/or focused on a conflict-affected population. This includes eHealth and teleradiology services delivered by remote providers as well as domestic health system provision. Categorisation of key themes emerging from this search was carried out to examine the manner in which medical imaging services are delivered in these settings, highlight key providers and determine gaps for further health system intervention.

Definitions of key terminology.

**Table 1:** Key terminology used throughout this paper and within radiology have been identified and defined

Terms	Definitions
Radiology	A branch of medicine that uses imaging technology to diagnose and treat disease
Medical Imaging	Several different technologies that are used to view the human body in order to diagnose, monitor, or treat medical conditions
Radiography	The process of taking radiographs, pictures, within radiology
X-ray Radiography	Ionising imaging modality using X-ray radiation to produce mages of the body’s internal structures.
Computer Tomography (CT)	Cross-sectional imaging modality that combines a series of X-rays to form detailed internal images of the body.
Ultrasound	An imaging modality that uses high-energy sound waves to look at tissues and organs inside the body.
Magnetic Resonance Imaging (MRI)	Non-ionising imaging modality used to create 3D cross-sectional images. Equipment is large, expensive, and take a longer time than other imaging modalities.
Picture Archiving and Communication System (PACS)	System which acquires, stores, transmits, and displays radiographs.

The literature search examined English language articles from using Ovid Medline, Ovid Global Heath, PubMed and Google Scholar. Key words used include radiology, CT, X-ray, MRI, ultrasound, conflict, battle, war, tele-radiology, medical imaging, and radiography. A simultaneous grey literature search was carried out via ReliefWeb, The WHO Global Health Observatory, the Enterprise search engine (UN) and the Google search engine. Data was extracted and coded according to key domains e.g. provider, geographic region, imaging modality, intervention population.

## Results

Conflict areas identified in the included papers spanned a variety of countries including Ukraine, Rwanda, Afghanistan, Iraq, Yemen, Lebanon, Bosnia, and Gaza.

Analysis of search results identified 3 key themes:

1. military provision during active conflict (Table 2),
2. pre-existing local services operating during war time (Table 3),
3. remote service adaptation (Table 4).

Category one: military provision during active conflict.

Category one sources.

**Table 2:** List of sources with references falling under category one, with the main content and location of each summarised

USA military hospitals: Paediatric radiology, Iraq, and Afghanistan [5]
Radiology equipment in military hospital, Afghanistan [6]
USA military hospital: CT scan for craniofacial trauma, Afghanistan [7]
UK military hospitals: FAST/ CT/ Ultrasound Radiology, Afghanistan [8]
Join Forces: FAST and CT Radiology, Afghanistan [9]
FAST Paediatric patients, Afghanistan [10]
USA military hospitals: Paediatric Emergency Department Radiology, Iraq, and Afghanistan [11]
USA military hospitals: explosive multiple casualty incident FAST CT XR, Iraq [12]
USA military hospital: portable X-ray, Iraq [13]
Australian Medical Support Force, Radiology service, Rwanda [14]

10 articles described on-the-ground radiology provisions provided by military groups – mainly from the United States of America, and mostly hospitals based in Afghanistan. All were peer-reviewed journal articles. Topics focused mostly on comparison of different imaging modalities, with only one focusing on equipment set up in a military hospital.

Category 2: pre-existing local services during war time.

Category two sources.

**Table 3:** List of sources with references falling under category two, with the main content and location of each summarised

Radiology screening, Gaza [15]
Radiology screening, Gaza [16]
Radiologist shortage, Iraq [17]
Training programme for radio-oncology, Iraq [18]
Radiologist and equipment shortage, Iraq [19]
Radiologist, equipment shortage, and radio-oncology, Iraq [20]
Radiology service usage, Lebanon [21]
Attack on hospitals, Syria [22]
Radiologist shortage, Yemen [23]
Radiologist shortage and targeting of hospital, Yemen [24]
Radiology adaptations, Ukraine [25]

10 articles included describe pre-existing local services during wartime, with 7 coming for peer-reviewed journal sources and others from humanitarian organisations like Doctors Without Borders, and radiology news websites. All bar one source from Ukraine, depicted severe radiologist and equipment shortages that were impacting patient care of civilians.

Category 3: remote service adaptation.

Category three sources.

**Table 4:** List of sources with references falling under category three, with the main content and location of each summarised

USA military hospital: teleradiology, Afghanistan [26]
USA military hospital: teleradiology, Bosnia [27]
Syrian American Medical Society Teleradiology relief group, Syria [28]
Teleradiology Relief Group, Syria [29]
Teleradiology Relief Group, Syria [30]
Tele-ICU and Radiology review, Syria [31]
Post-mortem Radiology reporting, UK [32]
Teleradiology review [33]

7 sources focused on tele-radiology, with 3 describing one effort in Syria organised by the Syrian American Medical Society. 2 focused on tele-radiology of military hospitals, and a further review of the use of teleradiology in military hospitals. Access to PACS was limited in hospitals in conflict zones and articles described the usage of social media such as WhatsApp and Facebook to share photos of scans with a world-wide community of consultant and trainee radiologists.

## Discussion

1) *Conflict-affected settings are seriously underserved by medical imaging services*.

Conflict regions typically have a pre-existing shortage of radiologists and equipment that is exacerbated by conflict activity. This is at odds to the demand of radiology during conflict; the Lebanon War in 1982 saw 80% of the wounded presenting to triage sent directly to the radiology department, with CT emerging as one of the most important diagnostic imaging modalities in war [21]. Nevertheless, issues such as violence against health-workers continue to deplete medical and radiological workforces, further compromising the provision of radiology services in already underserved settings.

Yemen’s Civil war began in 2014, though it was already suffering with acute shortages in radiology. In 2012, survey of 20 of the 55 General Government Hospitals found that 9 had no radiologists at all [23]. Similarly in 2003, before the Iraq War began there were 6 radiologists in Baghdad Teaching Hospital though it ideally needed 12 [17]. War exacerbated this shortage with just 2 remaining in Baghdad’s teaching hospital after 2011. Unpublished data from 2008 showed that of Baghdad’s 125 radiologist, 2% had been killed and 5% threatened, 25% emigrated, 30% left the capital to safer areas of Iraq, and 10% had changed careers. Senior health professionals including Professors of Radiology had been targeted by militants, tortured, and assassinated – over all physician specialities, 90% said they had been targeting while in clinics or their vehicles [17].

Postgraduate training was neglected in Iraq following their national health service receiving 90% cuts to funding since the 1990s, culminating in the Iraqi Committee for Medical Specialisation stopping Iraq’s only postgraduate radiology teaching program in 2005 [17]. In 2006, 60% of all physicians in Iraq had left the country with only 17% expressing an interest to return [20]. This provides future challenges for the reinstatement of programmes once due to a lack of senior physicians to provide training and mentorship. These conditions will likely contributing to long-standing and persistent radiological workforce shortages. In 2017, the Ministry of Health in Iraq found there were 76 Radiology physicians for 38 million population. A new board-certified training programme was set up in 2013 and 6 physicians have successfully completed as of 2019 [18] [20][21].

Aside from direct attacks on physicians and hospitals, sanctions and plummeting financial resources available to healthcare sector also provide challenges to the procurement of essential radiology equipment. In Iraq during before the Gulf war in 1989, health imports of medicine and medical equipment was US$500 million but fell to US$50 million in 1991 [20]. Nuclear medicine infrastructure such as radionuclide sources and generators were judged to be ‘dual use’ (i.e. capable of being used for beneficial as well as malign intent) and therefore their use prohibited by the United Nations [20] [19].Other technical constraints include erratic electricity supply due to damage to the national grid disrupting hospital infrastructure, including radiology specific tools in settings such as Iraq and Gaza [20, 34].

In Ukraine following the Russian Invasion on 24th February 2022, many radiologists remained in-country, taking on additional trauma caseloads on top of existing pressures [24]. Utilising social media, medical literature on the assessment of war wounds by medical imaging, were shared amongst colleagues [25]. Hospitals in Ukraine continue to actively encourage patients to continue routine screening such as mammographic screening for breast cancer [25]. Despite these efforts, it is unlikely that services will not experience significant disruption as the conflict continues to erode physical, human and financial health sector resources.

In contrast, the conflict in Gaza has spanned decades. The main clinical centre in Gaza, Al-Shifa hospital, conducted investigations on 254 Palestinian patients who had undergone war-related extremity amputation, finding 94 to be recommended for further radiological investigations. Each patient was sent for abdominal and chest CT, abdominal ultrasound, and MRI of amputation stump. However, 10% of data was missed due to unavailability of diagnostic equipment, the CT machine broke down twice, was prioritised for more urgent cases, and MRI investigations were limited due to metal shrapnel residue in patients’ stumps. Investigations found fatty liver infiltration, lung nodules, pathologically enlarged lymph nodes, atelectasis, liver lesions, and weaponry shrapnel in the chest, abdomen, scrotum and in the amputated and non-amputated limbs. Heavy metal residue from weaponry have been found in the wounds of Palestinians, and have been suggested to be linked to birth defects [15].

In some cases, there may be misdirection of funds to radiology services which do not serve the local population. Despite the acute shortage of wider radiology services and cancer treatment facilities, the Palestinian Ministry of Health has officially made mammography services their top priority, recommending annual scans for women over 50 [16]. Gaza and the West Bank already have 39 mammographic machines with 90% non-functioning or underutilised due to staff shortages [16]. Some have been at great cost, with international organisations supplying a digital mammographic machine for $130,000 USD in 2017 [16]. This focus on mammography services is likely due to the poor-quality research undertaken within Gaza which exaggerates the benefits, with papers detailing high rates of false positive results left unpublished [16]. With around 50% of these machines in the private or non-governmental sectors [16], it should be considered that financial incentive might be a factor.

2) *Services that exist are largely centred in military organisations*.

The emphasis on military provision of radiological services is a consequence of these organisations being generally well-resourced, adaptable, able to carry out remote work and capable of securing radiation infrastructure in conflict-affected territories.

A scaled down military combat support hospital in Afghanistan in 2002 was equipped with 18 medical/ surgical beds, 10 intensive care units and 2 operating rooms, carrying out 500 diagnostic images monthly [7]. Radiology departments deployed by military forces are well staffed with radiologist and technicians. In Iraq 2008 in US Airforce Theatre Hospital, there were 3 radiologists (2 were back-up) and 6 radiology technicians while UK-run military hospitals in Iraq/ Afghanistan 2009 employed 2 consultant radiologists and 6 radiographers [8, 12].

Much of the literature highlighted the frequent use of CT scanning and how this modality provided essential to properly triage causalities. In Afghanistan in 2002 for example, a American military hospital found CT scanning to be an essential capability for the care of craniofacial trauma patients to determine those requiring neurosurgical intervention [7].

Following 3 incidents in Iraq 2008 arising from the use of improvised explosive devices (IEDs), 46 of the 50 living patients presenting to the US Airforce Theatre Hospital received radiological imaging, with 90% receiving CT scanning. 93% of all CT scans formed part of a trauma scan, and 49% of all CT scans were deemed clinically significant. Other modes of radiology were used with x-rays were performed on 70% of patients, and FAST/ EFAST examinations on 38% of all patients to ensure unstable patients were prioritised for imaging [12].

A multidetector CT significantly increased the scope of treatment for trauma patients and prevented unnecessary exploratory surgery in the UK military hospitals of Iraq and Afghanistan 2009, with patients imaged and reported by a consultant radiologist within an hour of arrival [8]. Abdominal CT scanning in Afghanistan 2012 at Joint Force Camp Bastion Military Hospitals found a higher sensitivity, specificity, positive predictive value, negative predictive value, and accuracy when compared to FAST in trauma systems [9]. Similarly for paediatric patients with penetrating abdominal injuries, CT scans had a higher sensitivity and identification of free fluid compared to FAST [10]. However, 45% of paediatric patients presented with another patient and 30% with another injured child affording FAST an essential role in mass casualty scenarios requiring triage if rapid access to CT is unfeasible [10].

In all, between January 2007 and January 2016 in Afghanistan and Iraq paediatric patients accounted for 8% of all trauma admissions and 12,376 imaging studies were conducted on 2920 children (84.9% of all paediatric presentations) – 49.8% of which CT scans. A high proportion of CT exams amongst penetrating injury patients suggesting healthcare professional’s preference to defer exploratory laparotomy procedures, however FAST examinations were undertaken only 36.9% of paediatric presentations potentially attributed to it being omitted in those patients that met guidelines for laparotomy [11]. Only 1.3% of all CT scans amongst all populations in the time period underwent CT angiography, suggesting it was not routinely utilised in vascular injuries amongst both adults and children [11].

Military hospitals are well-resourced to manage the hostile conditions of conflict. For example, utilising high-efficiency air-filters and placing CT scanners in a restricted access isoshelter to combat dust, and air conditioning units to keep CT scanners cool in hot climates. However, like local hospitals, erratic electricity and power outages cause hard drives to require reformatting and result in lost studies [6]. Historical examples of forward operating bases in Iraq in 1992 showed the value of a portable dental x-ray being in the forward-facing tent, 30 yards ahead of the standard x-ray equipment tent. This allowed limb x-rays to occur in the initial tent, and prioritised transfer for those with major and abdominal injuries [13].

Teleradiology, first used in Operation Desert Storm between Saudi Arabia and USA via satellite, has an increasing role to play in military radiology [27]. In 1995, CT scanners were deployed to USA military hospitals with a maintenance team, but without an accompanying radiologist, and instead over the duration of the conflict 10,000 x-rays, CT scans and Ultrasounds were interpreted by a radiologist deployed in Germany/ Hungary [33].

While this prevented costly unnecessary evacuations, which would have required 4 vehicles, 8 combat soldiers or two helicopters, the challenge of image quality, appropriate utilisation, and radiation dose management due to the lack of an onsite radiologist did limit the overall maximum effectiveness of the intervention [33]. In Iraq and Afghanistan, the USA military continued to utilise teleradiology with CT scanners deployed to each unit of a combat support hospital, which has its own wide area of geographic responsibility, whilst deploying radiologist to only one of these with equipped with networked PACS provision.

A high band-with satellite link between Bagram Afghanistan and Landstuhl Regional Medical Centre Germany (theatre support hospital) was established in 2002, using a brigade remote subscriber system providing mobile communication capability via commercial switches and routers, that allowed the transmission of high-resolution studies for second opinions [26].

Aside from equipment, the physician workforce brought in by military hospitals provides opportunity for humanitarian work for the underserved local population. Following the Rwandan Civil War, Australian Medical Support Forces provided the first specialist x-ray services in the aftermath of the war [14]. USA military radiologists in Afghanistan providing humanitarian paediatric care encountered a variety of presentations including gastrointestinal infection, anthrax, and tuberculosis [5]. Delayed presentation of congenital diseases such as Hirschsprung disease, Ewing sarcoma and retinoblastoma were common due to limited access of local healthcare [5]. Paediatric patients admitted to USA military Hospitals in contributed to 25% of hospital bed days in Afghanistan and 10–12% in Iraq, with 75% of these admissions due to traumatic injuries [5].

3) *Innovation in radiology provision can provide temporary solutions to acute service gaps*.

The Syrian American Medical Society established a Teleradiology Relief Group (TRG) in February 2015 when just one radiology resident was serving a population of 400,000 without MRI scanners or PACs, though there were multiple portable ultrasound scanners, x-ray machines and one 4 slice CT scanner [28]. The TRG was composed of 4 volunteer board-certified radiologists and 2 volunteer residents across a 24-time zone [28]. As of January 2018, the TRG interpreted 497 radiological examinations, of which 75% were CT scans and 24% plain films [28]. 97% of the CT scans were without contrast due to its high cost and shortage, which proved to be the biggest challenge the TRG reported as trauma scans were consequently difficult to interpret [28, 29]. Radiological examinations were shared using satellite internet via Facebook, WhatsApp, and Telegram Social Media Groups as DICOM (57%) and JPEG (43%) images, although this method of sharing was challenged by poor cell phone camera quality [28, 29]. Additional challenges included scans obtained with poor quality equipment, or by untrained individuals producing improperly projected films, CT images printed on viewing boxes, or difficult to interpret ultrasound series [29, 30]. Despite this, preliminary reports were generated in 24 h by a TRG resident which was then confirmed by the attendings [28].

Similar to the TRG group, a tele-ICU program was set up, modelled on pre-existing programs in the USA [31]. 20 volunteer intensivist physicians across Europe and North America provided remote care, including radiology reviews, to 5 civilian ICUs in Syria using free social media apps and mobile phone cameras [31]. The implementation of an Electronic Medical record in August 2015 allowed direct viewing of radiology images if the images were obtained by digital technology, thereby reducing some of the difficulties experienced by the TRG group, however, some radiographs were still performed using hard-copy films in which cases uploading of mobile camera photos was still necessary [31]. Implementation of EMR was slow due to user training, limited bandwidth making the application slow, and it meaning nurses had to leave the bedside to upload their care plan [31].

### Key recommendations

The immediate challenge facing local services is lack of radiologists and high-quality equipment that can be operated correctly by trained staff, in addition to power cuts and sanctions. Retaining existing radiologists is essential to ensure care for the local population, and efforts should be made to keep hospitals and health care professionals safe from targeted attacks. More immediately, radiological equipment is often in short supply due to cost and damage, leading to poor quality films, which are hindered further by the restricted availability of contrast media due to international sanctions. It is imperative funding is carefully targeted to the most required and evidence-based services. Laptops or good quality camera smart phones should also be considered for donation. This will enable more humanitarian outreach physicians abroad to set up programs such as the TRG to provide radiology care midst the radiology shortages.

Military hospitals indicate heavy use of CT scanners, though while still utilising other modalities such as ultrasound for FAST in triage situations. Military hospitals should ensure CT scanners are adapted to the conditions of the conflict environment – dust, temperature, electricity supply – from the get-go and flanked with maintenance teams to ensure good images [33]. Further due to the frequent use of CT scans on paediatric patients, military radiologists/ radiology technicians should be trained on paediatric radiology and the conditions commonly seen in underserved populations.

Military hospitals and local services both benefit from teleradiology. Teleradiology in conflict zones must utilise slow satellite link connection dependent on bandwidth in the absence of fibreoptic networks [33, 32].Technology adapted to the use of satellite link needs to be improved to increase transmittance speed of radiological scans.

Further advancements in technology, such as the integration of artificial intelligence (AI) may also improve service. As described by the Lancet Commission, AI may provide a role in aiding the operator during point of care ultrasound, to the use of algorithms to aid triage and patient flow [2]. However, AI is currently not widely implemented within radiology or wider healthcare delivery. Therefore, following further development of this technology, it would need to be trialled and its effectiveness determined. Due to the infrastructure challenges within conflict zones, AI may prove a greater hinderance.

To improve the delivery of radiology in conflict zones, further research into the experience of radiologists and their patients should be conducted. This is difficult owing to the nature of instability in these areas; however, this effort could be supported via the global community of radiologists health systems researchers [31].

### Study limitations

Studies were only included if they were in the English Language. Additionally, many conflict zones are not in English speaking regions. Therefore, many sources, in particular grey literature, may not appear in searches due to being written in other languages.

## Conclusion

Radiology is an essential healthcare service in conflict zones. Not only is it vital for assessing injuries inflicted directly from conflict, but also for the diagnosis and treatment of wider disease.

Medical professionals, radiologists, who are integral to the capture, interpretation, and reporting of medical imaging studies are essential for delivering radiology services. However, local populations are severely underserved due to pre-existing and subsequent worsening of radiologist shortages during conflict. Military hospitals or programmes may offer some care to civilians – with these tending to be the primary services that exist during certain conflicts. Importantly, many military hospitals experience a significant burden of paediatric presentations but may not be adapted well for these patients. Further, in long-standing conflict, the population may have even further demand on radiology services with complex presentations owing to sustained underserving of healthcare in the region.

Radiology has further challenges owing to the specialist equipment required. Not only do these tend to be large and expensive, but they are often sensitive to their environment. Therefore, challenges include transportation of the equipment, ensuring they run effectively despite unstable conditions such as power outages, adapting machines to harsh climates, and finally the complexity of sourcing proscribed materials such as contras media. Many contrasts that are key for producing diagnostic studies may be banned or heavily restricted by sanctions placed on conflict territory or related border. Furthermore, due to the expense of necessary machinery these machines are often in short supply, or may be outdated in. Military hospitals may face fewer of these obstacles due to less significant resource constraints than public or NGO services.

Reviewing the experiences of radiologists in areas of conflict provides insight into these challenges, and their potential solutions. However, this information is not easy to access as it is dispersed across multiple sources and languages. An effort should be made by the to document the delivery of radiology services in conflict-affected settings to allow innovation for future scenarios. Current literature however has revealed three key areas of recommendation: the availability of good quality equipment and radiologists, military hospitals adapted to the terrain of the conflict and with staff knowledgeable of the complex health of the local population, and finally the growing importance of tele-radiology.

## Data Availability

Not applicable.

## Abbreviations

CT: Computed Tomography
MRI: Magnetic Resonance Imaging
PET: Positron Emission Tomography
SPECT: Single Positron Emission Tomography
PACS: Picture Archiving and Communication System
TRG: Teleradiology Relief Group
